# Three years evaluation of peritoneal dialysis and hemodialysis absorption costing: perspective of the service provider compared to funds transfers from the public and private healthcare systems

**DOI:** 10.1590/2175-8239-JBN-2021-0118

**Published:** 2022-01-19

**Authors:** Alyne Schreider, Celso Souza de Moraes, Natália Maria da Silva Fernandes

**Affiliations:** 1 Universidade Federal de Juiz de Fora, Faculdade de Medicina, Instituto de Ciências Biológicas, Juiz de Fora, MG, Brasil; 2 Universidade Federal de Juiz de Fora, Faculdade de Administração e Ciências Contábeis, Juiz de Fora, MG, Brasil.

**Keywords:** Costs and Cost Analysis, Health Expenditures, Unified Health System, Supplemental Health, Peritoneal Dialysis, Renal Dialysis, Custos e Análise de Custos, Gastos em Saúde, Sistema Único de Saúde, Saúde Suplementar, Diálise Peritoneal, Diálise Renal

## Abstract

**Introduction:**

72% of renal replacement therapy (RRT) clinics in Brazil are private. However, regarding payment for dialysis therapy, 80% of the patients are covered by the Unified Health System (SUS) and 20% by private healthcare (PH).

**Objectives:**

To evaluate costs for peritoneal dialysis (PD) and hemodialysis (HD) from the perspective of the service provider and compare with fund transfers from SUS and private healthcare.

**Methods:**

The absorption costing method was applied in a private clinic. Study horizon: January 2013 - December 2016. Analyzed variables: personnel, medical supplies, tax expenses, permanent assets, and labor benefits. The input-output matrix method was used for analysis.

**Results:**

A total of 27,666 HD sessions were performed in 2013, 26,601 in 2014, 27,829 in 2015, and 28,525 in 2016. There were 264 patients on PD in 2013, 348 in 2014, 372 in 2015, and 300 in 2016. The mean monthly cost of the service provider was R$ 981.10 for a HD session for patients with hepatitis B; R$ 238.30 for hepatitis C; R$197.99 for seronegative patients; and R$ 3,260.93 for PD. Comparing to fund transfers from SUS, absorption costing yielded a difference of -269.7% for hepatitis B, +10.2% for hepatitis C, -2.0% for seronegative patients, and -29.8% for PD. For PH fund transfers, absorption costing for hepatitis B yielded a difference of -50.2%, +64.24% for hepatitis C, +56.27% for seronegative patients, and +48.26 for PD.

**Conclusion:**

The comparison of costs of dialysis therapy from the perspective of the service provider with fund transfers from SUS indicated that there are cost constraints in HD and PD.

## Resumo

**Introdução:**

72% das clínicas de terapia renal substitutiva (TRS) no Brasil são privadas. Entretanto, quanto ao pagamento da terapia dialítica, o Sistema Único de Saúde (SUS) cobre 80% dos pacientes e, a saúde privada (SP), 20%.

**Objetivos:**

Avaliar custos de diálise peritoneal (DP) e hemodiálise (HD) na perspectiva do prestador de serviços, comparando com repasses do SUS e saúde suplementar.

**Métodos:**

O método de custeio por absorção foi aplicado em clínica privada. Horizonte de estudo: Janeiro 2013 - Dezembro 2016. Variáveis analisadas: pessoal, suprimentos médicos, despesas tributárias, ativos permanentes, benefícios trabalhistas. Utilizou-se para análise o método da matriz de *input-output*.

**Resultados:**

Realizou-se um total de 27.666 sessões de HD em 2013, 26.601 em 2014, 27.829 em 2015, e 28.525 em 2016. Havia 264 pacientes em DP em 2013, 348 em 2014, 372 em 2015, e 300 em 2016. O custo médio mensal do prestador de serviços foi R$ 981,10 por sessão de HD para pacientes com hepatite B; R$ 238,30 para hepatite C; R$ 197,99 para pacientes soronegativos; R$ 3.260,93 para DP. Em comparação com repasses do SUS, o custeio por absorção mostrou uma diferença de -269,7% para hepatite B, +10,2% para hepatite C, -2,0% para pacientes soronegativos, e -29,8% para DP. Para repasses da SP, o custeio por absorção para hepatite B mostrou uma diferença de -50,2%, +64,24% para hepatite C, +56,27% para pacientes soronegativos, e +48,26 para DP.

**Conclusão:**

A comparação de custos da terapia dialítica da perspectiva do prestador de serviços com os repasses do SUS indicou que existem restrições de custos em HD e DP.

## Introduction

The International Society of Nephrology^1^ emphasized in a recent publication that chronic kidney disease (CKD) affects 850 million people worldwide and is one of the main contributors to the global burden from chronic non-communicable diseases (NCDs). CKD is both a cause and a consequence of NCDs and is the main cause of catastrophic health expenditure. If not addressed, it is projected that by 2040 CKD will be the fifth most common cause of years of life lost.

The 2018 Brazilian dialysis census reported 133,464 patients on dialysis therapy (DT) in Brazil^2^. The Brazilian public health system, called the Unified Health System (*Sistema Único de Saúde* - SUS) was implemented in 1990 as a result of the last constitutional modification in 1988^3^. With regard to private healthcare (PH), the Brazilian National Supplementary Health Agency (*Agência Nacional de Saúde Suplementar*) established in 1999 the rules for private health care plans and implemented basic guarantees for beneficiaries^4^. Of the clinics providing renal replacement therapy (RRT) in Brazil, 72% are private, 9% are public, and 19% are philanthropic. Regarding DT payment, 80% of patients are covered by the SUS and 20% by PH^2^.

Different methods are used in cost studies used, and even when similar methods are used, the description of the results is not standardized^5^. There is a great variability in costs between countries and even between dialysis centers. In general, such studies take the perspective of the payer^6^.

In this study, we were particularly interested in the perspective of the DT private service provider because, to the best of our knowledge, no studies have compared the top-down (payer) and bottom-up (service provider) perspectives of in Brazil^6^. One in Finland^7^ compared the costs of hemodialysis (HD), continuous ambulatory peritoneal dialysis (CAPD), and renal transplantation (Tx) from the perspective of the service provider, concluding that there was no difference between HD and CAPD costs whereas Tx costs were significantly less than other methods after the first six months. A Turkish study^8^ published in 2004 compared three dialysis units from the same perspective and analyzed the costs of HD, CAPD, and Tx. The authors observed that Tx had a higher cost in the first year, but thereafter the costs were less than those for CAPD or HD. Another study^9^ comparatively evaluated costs in several European countries from the perspective of the service provider and concluded that cost studies from that perspective made the data more consistent, transparent, and comparable. Finally, in 2015, a study^10^ conducted in the UK from the perspective of the service provider performed a cost-effectiveness analysis comparing high-dose HD and conventional HD and found that high-dose HD was more cost-effective.

In Brazil, there are a few studies on the cost of DT. In general, they are conducted from the payer's perspective and their results are not consistent. Of those that compared the costs of HD and peritoneal dialysis (PD), two reported a lower cost for PD^11^^,^ 12 and two reported a lower cost for HD^13^^,^ 14. The study that included Tx^12^ had similar results to studies around the world, concluding that Tx has a lower cost than DT from the second year onward.

There are no cost studies on DT from the perspective of the service provider using the absorption costing method suggested by the Brazilian Ministry of Health (MoH)^15^. The costing method defines how the cost evaluation of a given product was made, and this helps to define how and to which costs the products should be allocated. According to the MoH Cost Management Introduction Manual, there are the following costing methods: absorption cost, full costing, marginal cost, direct costing, variable costing, and activity-based costing. Absorption costing considers all production costs of a product, whether direct or indirect, fixed or variable, structural or operational. Thus, both variable costs (which only appear when each unit is manufactured) and fixed costs (which are independent of each unit and relate to production conditions) are integrated into the book value of the manufactured product. It is a method generally accepted and recognized by accountants, auditors, and tax legislation. Therefore, it is recommended by the public sector and the MoH to strengthen the legal and managerial area of hospital cost management, due to its methodological rigor^15^. It is believed that DT cost studies from the perspective of the service provider produce data that is more consistent, transparent, and comparable. Therefore, the aim of the present study was to evaluate the absorption costing of PD and HD from the point of view of the service provider (bottom-up) and to compare the cost data with those resulting from funds transfered from the public (SUS) and private (PH) healthcare systems (top-down).

## Methods

The study followed the best practices of the Accountant's Code of Professional Ethics, Brazilian Accounting Standards No. 1 of February 7, 2019, and article 7 of the Brazilian Financial Law, Law No. 13,709 of August 14, 2018. The owner gave consent for the confidential data to be used for research and teaching purposes, provided that they were anonymized according to the law. The data were collected by a trained and licensed accountant.

A retrospective cohort study was conducted from the perspective of the service provider, and data were compared from that of funds transferred from SUS and PH. The time frame of the study was January 2013 to December 2016. The method used was absorption costing, a method suggested by the MoH^15^. The study was conducted in a private clinic providing services in Juiz de Fora, including care to patients with CKD who are on conservative treatment, HD, PD, and pre- and post-renal transplant outpatient care, through SUS and PH. The capacity of HD is 35 patients per shift, with a specific room for HD patients with hepatitis B. The clinic follows the norms of the Board of Directors Resolution (National Health Surveillance Agency) of March 14, 2014^16^ regarding organization, patient care, physical infrastructure, dialyzers, arterial and venous lines, equipment and materials, and water quality, in addition to microbiological analyses of dialysate. These standards apply to all RRT service providers in Brazil, whether public or private.

The variables included in the study apply to all Cost Centers (CCs), defined as departments classified according to the activity they perform, in accounting spreadsheets. In this study, CCs were divided into 13 groups according to the activity performed. The variables could be included in one or more CCs.

The variables included were: clinic receipts - SUS and PH receipts, all proceeds were included in the receipts, from dialysis therapy itself and from procedures; Costs with permanent assets - acquisition of assets (HD machine), furniture and fixtures, machinery and equipment, and parts for HD machines; Costs with human resources - benefits such as food stamps, loans for employees and interns, vacations, Employment Compensation Fund (ECF), board medical fees, overtime, income taxes, National Social Security Institute (NSSI), resident doctors, uniforms, health and dental plans, contract terminations, salaries, medical services and union; Medical material costs - dialysis accesses, fistula needles, PD bags, citralocks, dialysis concentrates, dialyzers, personal equipment materials, disposable gloves, heparin, dialyzer lines, materials for water treatment, medications, oxygen and compressed air, sanitizing and physiological solutions; Costs with other expenses -Sterilized Material Center (SME), meals for patients, expenses with waste treatment, cell phones, telephone lines, internet use, water supply, electricity, software maintenance, building maintenance and installations, computer materials, insurance, taxi, vans and parking lots, freight and carts, snacks and meals, laundry, rental of HD machines (3 machines), maintenance of various equipment, pantry, office and cleaning supplies, accounting and legal services, fast delivery services, printing services, outsourced laboratory services and outsourced corporate services; Costs with tax expenses - Contribution to Social Security Financing (COFINS), Social Contribution to Net Income (CSSL), Service Tax (ISS) (deposit in court), Service Tax of Any Nature (ISSQN), municipal ISSQN, National Social Security Institute (NSSI) (service invoice), Urban Land and Property Tax (IPTU), Corporate Income Tax (IRPJ) (quarterly), other taxes and fees and the Social Integration Program (PIS).

To evaluate personnel activity, all processes in each business unit were mapped using the time, event, space, and person instrument (TEvEP). The TEvEP is a standard tool used for accounting appraisals and evaluates the professional who performs the activity, the time spent on the activity, the location where it is performed, and the interrelationship between TEvEPs. A total of 47 TEvEPs were performed, and interviews were conducted to build a matrix. These data are presented per CC or business unit as the percentage of time spent by each professional in that event and in that unit.

When an absorption costing study is conducted, revenues are not considered. Thus, expenses, costs, and investments were the amounts considered. Revenue data were included to compare the costs with funds transferred from the SUS and PH. Losses were disregarded; in theory, losses should be considered, but in this case they were insignificant. For example, a batch of defective dialysis lines is considered a loss, but as this did not occur, loss was negligible. Another relevant factor is that expenditures with permanent assets are included in the cost. An important difference in the treatment of these data is between that made by an accountant and that made by an economist: the economist would not consider the permanent assets, whereas the accountant aggregates these permanent assets in the absorption costing in order to evaluate the overall cost. An analysis done this way provides a better view of the whole.

**Figure 1:**
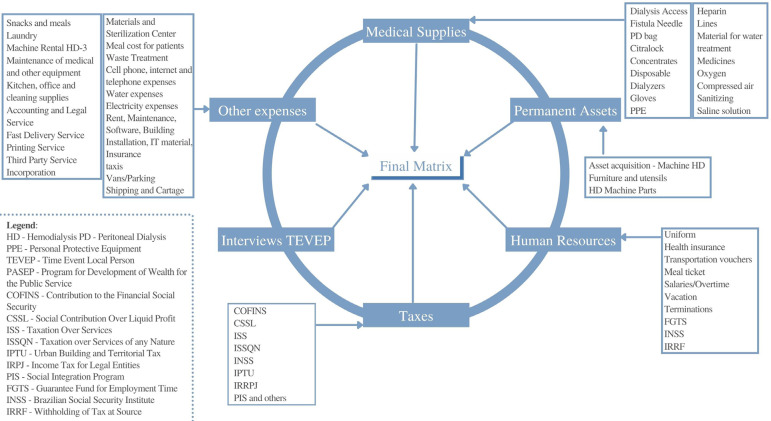
Flowchart of data collection and assembly of the final matrix.

Figure 1 contains a flowchart showing the order in which the data were collected so that final matrix is logically constructed.

### Data analysis

There is a difference between the economic and accounting approaches to health costs. Because health professionals are not familiar with these concepts, it is necessary to conceptualize basic differences between these two approaches. Economics is a social science that studies the production, distribution and consumption of assets and services. Accounting has its own object - the equity of companies - and consists of knowledge obtained by rational methodology, with the conditions of generality, certainty, and search for causes, at a qualitative level like the other social sciences^15^.The main method used by the accounting approach is absorption costing, which is the method suggested by the MoH^15^.

After the data were collected and compiled in Excel spreadsheets, input-output matrix analysis was done based on the description of Wassily Leontief^17^.This analysis is used to calculate costs when there is a need to incorporate multiple variables in addition to the direct cost of the product, such as rates, time required to perform tasks, etc. Currently, a calculation derived from the initial idea of Wassily Leontief is the calculation of the reciprocal matrix^18^.

## Results

From 2013 to 2016, there were no adjustments in the payment by SUS for HD or PD sessions.

A total of 27,666 HD sessions were performed in 2013, 26,601 were performed in 2014, 27,829 were performed in 2015, and 28,525 were performed in 2016.

Table 1 shows the main reciprocal matrix that contains the cost centers. These centers can be directly or indirectly linked to the final product (dialysis therapy). As the matrix is reciprocal, the same variables are shown in rows and columns. Matrix calculations are shown in the supplementary material. The auxiliary and inverse matrixes (essential part of matrix calculation) are shown in Tables 2 and 3. Finally, Table 4 shows the comparison between the cost in the dialysis unit and the funds transferred from the SUS and PH, adjusted by the consumer price index. For data that were related to more than one cost center, apportionment criteria were used, which is also shown in the supplementary material. The calculations summarized above are a simplified way of referring to accounting calculations.

**Table 1:** Principal matrix

Principal matrix (%)																			
Variable	Administrative	Social Security	Nursing	ETA	Financial	Hygiene/cleaning	Nutrition	Psychology	Reuse	Sec.- Outpatient	Secr. - Assist.	Secr. -HD	Supplies	Outpatient - Nephropathies	Outpatient -Transplantation	PD	HD (yellow room)	HD (white room)	Hospital HD
Administrative	**49.85**	0.67	0.49	1.10	0.45	0.45	0.67	0.67	0.45	0.45	0.45	0.45	16.78	21.57	0.45	0.48	0.47	3.46	0.65
Social Security	0.13	**31.19**	0.13	0.13	0.13	0.13	0.13	0.13	0.13	0.13	0.13	0.13	0.13	0.13	28.75	0.91	0.34	36.54	0.53
Nursing	0.00	0.00	**2.30**	0.41	0.00	0.04	0.00	0.00	0.16	0.00	0.00	0.00	0.00	0.00	3.86	1.42	1.11	78.08	12.63
WTS	0.00	0.00	0.00	**41.97**	0.00	0.00	0.00	0.00	0.05	0.00	0.00	0.00	0.55	0.00	13.25	0.00	0.17	28.55	15.46
Financial	0.02	0.00	0.43	0.00	**34.18**	0.00	0.00	0.00	0.00	0.00	0.00	0.00	0.00	0.00	11.57	0.52	0.27	47.27	5.72
Hygiene/cleaning	2.14	1.91	0.11	0.34	2.37	**1.49**	1.91	2.14	4.10	2.60	0.34	3.63	2.68	1.68	1.91	3.64	8.09	55.56	3.34
Nutrition	0.00	0.00	0.00	0.00	0.00	0.00	**13.69**	0.00	0.00	0.00	0.00	0.00	0.00	0.00	0.00	0.94	0.49	84.87	0.00
Psychology	0.00	0.00	0.00	0.00	0.00	0.00	0.00	**26.04**	0.00	0.00	0.00	0.00	0.00	0.00	34.49	0.00	0.22	37.50	1.75
Reuse	0.00	0.00	0.00	0.00	0.00	0.00	0.00	0.36	**25.41**	0.00	0.00	0.00	0.00	0.00	0.00	0.00	0.00	74.24	0.00
Secretarial - Outpatient	0.00	0.00	0.00	0.00	0.00	0.00	0.00	0.00	0.00	**10.37**	0.00%	0.00	0.00	20.57	68.88	0.00	0.00	0.00	0.18
Secretarial - Assist.	0.00	0.00	0.05	0.00	2.90	0.00	0.00	0.00	0.00	0.00	**1.21**	0.00	0.00	0.00	0.00	1.90	0.54	93.39	0.00
Secretarial - HD	0.00	0.00	0.73	0.00	0.00	0.00	0.00	0.00	0.00	0.00	0.00	**4.75**	0.00	0.00	0.00	0.00	0.54	93.97	0.00
Supplies	0.23	0.23	0.23	0.23	0.23	0.23	0.23	0.23	0.23	0.23	0.23	0.23	**50.62**	0.23	0.23	0.62	0.43	41.77	3.36

WTS: water treatment station; PD: peritoneal dialysis; HD: hemodialysis

**Table 2:** Auxiliary matrix and inverse matrix

Auxiliary matrix (%)																	
Administrative	**50.15**		-0.13	0.00		0.00	-0.02		-2.14	0.00		0.00	0.00	0.00	0.00	0.00	-0.23
Social Security	-0.67		**68.81**	0.00		0.00	0.00		-1.91	0.00		0.00	0.00	0.00	0.00	0.00	-0.23
Nursing	-0.49		-0.13	**97.70**		0.00	-0.43		-0.11	0.00		0.00	0.00	0.00	-0.05	-0.73	-0.23
WTS	-1.10		-0.13	-0.41		**58.03**	0.00		-0.34	0.00		0.00	0.00	0.00	0.00	0.00	-0.23
Financial	-0.45		-0.13	0.00		0.00	**65.82**		-2.37	0.00		0.00	0.00	0.00	-2.90	0.00	-0.2%
Hygiene/cleaning	-0.45		-0.13	-0.04		0.00	0.00		**98.51**	0.00		0.00	0.00	0.00	0.00	0.00	-0.23
Nutrition	-0.67		-0.13	0.00		0.00	0.00		-1.91	**86.31**		0.00	0.00	0.00	0.00	0.00	-0.23
Psychology	-0.67		-0.13	0.00		0.00	0.00		-2.14	0.00		**73.96**	-0.36	0.00	0.00	0.00	-0.23
Reuse	-0.45		-0.13	-0.16		-0.05	0.00		-4.10	0.00		0.00	**74.59**	0.00	0.00	0.00	-0.23
Secretarial - Outpatient.	-0.45		-0.13	0.00		0.00	0.00		-2.60	0.00		0.00	0.00	**89.63**	0.00	0.00	-0.23
Secretarial - Assist.	-0.45		-0.13	0.00		0.00	0.00		-0.34	0.00		0.00	0.00	0.00	**98.79**	0.00	-0.23
Secretarial - HD	-0.45		-0.13	0.00		0.00	0.00		-3.63	0.00		0.00	0.00	0.00	0.00	**95.25**	-0.23
Supplies	-16.78		-0.13	0.00		-0.55	0.00		-2.68	0.00		0.00	0.00	0.00	0.00	0.00	**49.38**

Inverse matrix (%)														2013	2014	2015	2016
Administrative	199.77	0.40	0.00	0.01	0.07	4.38	0.00	0.00	0.00	0.00	0.00	0.00	0.95	203,906.04	240,907.93	241,586.46	206,440.47
Assist. Social	2.19	145.34	0.00	0.01	0.00	2.89	0.00	0.00	0.00	0.00	0.00	0.00	0.70	74,795.81	80,697.00	89,279.01	86,982.59
Nursing	1.19	0.21	102.35	0.00	0.68	0.21	0.00	0.00	0.00	0.00	0.07	0.79	0.49	66,298.65	49,187.09	64,602.23	61,703.98
WTS	4.08	0.35	0.72	172.33	0.01	0.72	0.00	0.00	0.00	0.00	0.00	0.01	0.82	167,760.28	160,879.72	166,778.86	192,524.25
Financial	1.69	0.32	0.00	0.01	151.93	3.74	0.00	0.00	0.00	0.00	4.47	0.00	0.75	181,093.63	215,406.13	204,031.14	225,523.52
Hygiene/cleaning	1.07	0.20	0.04	0.00	0.00	101.56	0.00	0.00	0.00	0.00	0.00	0.00	0.48	167,462.73	185,877.55	233,659.20	217,185.93
Nutrition	1.75	0.23	0.00	0.01	0.00	2.31	115.86	0.00	0.00	0.00	0.00	0.00	0.56	136,657.87	166,864.43	161,780.93	181,785.68
Psychology	2.05	0.28	0.00	0.01	0.00	3.03	0.00	135.22	0.64	0.00	0.00	0.00	0.65	61,165.02	78,717.61	97,069.38	104,252.62
Reuse	1.48	0.28	0.22	0.11	0.00	5.64	0.00	0.00	134.06	0.00	0.00	0.00	0.66	335,348.79	338,201.42	329,315.74	297,313.71
Secretarial - Outpatient	1.21	0.23	0.00	0.01	0.00	2.99	0.00	0.00	0.00	111.57	0.00	0.00	0.54	58,189.63	67,899.45	71,839.58	104,064.20
Secretarial - Assist.	1.07	0.20	0.00	0.00	0.00	0.39	0.00	0.00	0.00	0.00	101.22	0.00	0.48	31,309.81	35,166.05	29,571.59	43,052.96
Secretarial - HD	1.15	0.22	0.00	0.00	0.00	3.91	0.00	0.00	0.00	0.00	0.00	104.99	0.51	90,962.40	107,939.30	153,394.90	173,539.32
Supplies	68.00	0.54	0.01	1.93	0.02	7.03	0.00	0.00	0.00	0.00	0.00	0.00	202.88	226,231.13	247,989.58	268,441.45	379,704.89

WTS: water treatment station; HD: hemodialysis

**Table 3:** Result of the initial calculation (analysis of the procedures × funds transferred from SUS in the study period)

Procedures	2014	Vertical (%)	2015	Vertical (%)	Horizontal (%)	2016	Vertical (%)	Horizontal (%)	Cumulative horizontal (%)
PD	2,626.57	-12.1	2,720.28	-16.1	3.6	3,192.09	-27.1	17.3	21.5
PD - PRP	2,650.83	-13.1	2,747.39	-17.3	3.6	3,232.67	-28.7	17.7	21.9
DP - SUS transfer	2,342.81	100.0	2,342.81	100.0	0.0	2,511.49	100.0	7.2	7.2
HD - patients with B hepatitis	784.87	-338.4	866.88	-226.6	10.4	915.22	-244.8	5.6	16.6
HD - patients with B hepatitis - PRP	788.19	-340.3	870.75	-228.1	10.5	919.77	-246.5	5.6	16.7
HD - patients with B hepatitis - SUS transfer	179.03	100.0	265.41	100.0	48.2	265.41	100.0	0.0	48.2
HD - patients with C hepatitis	188.10	-5.1	208.93	21.3	11.1	227.00	14.5	8.6	20.7
HD - patients with C hepatitis - PRP	191.43	-6.9	212.79	19.8	11.2	231.55	12.8	8.8	21.0
HD - patients with C hepatitis -SUS transfer	179.03	100.0	265.41	100.0	48.2	265.41	100.0	0.0	48.2
HD - seronegative patients	162.20	9.4	172.14	3.8	6.1	183.19	-2.3	6.4	12.9
HD - seronegative patients - PRP	165.53	7.5	176.00	1.7	6.3	187.74	-4.9	6.7	13.4
HD - seronegative patients - SUS transfer	179.03	100.0	179.03	100.0	0.0	179.03	100.0	0.0	0.0
HD In-hospital	463.44	-74.6	466.95	-75.9	0.8	472.21	-77.9	1.1	1.9
HD In-hospital - PRP	480.63	-81.1	486.31	-83.2	1.2	492.94	-85.7	1.4	2.6
HD In-hospital - SUS transfer	265.41	100.0	265.41	100.0	0.0	265.41	100.0	0.0	0.0

DP: diálise peritoneal; PLR: participação nos lucros recebidos; HD: hemodiálise; SUS: Sistema Único de Saúde

**Table 4:** Comparison between cost in the unit and funds transferred from the SUS and social security, adjusted by the consumer price index

Procedures	Mean cost	SUS transfer 11/2018	%	SS transfer (1 health insurance plan)	%
PD	3,260.93	2,511.49	-29.8	4,834.69	48.26
HD - patients with B hepatitis	981.10	265.41	-269.7	392.10	-50.21
HD - patients with C hepatitis	238.30	265.41	10.2	392.10	64.54
HD – seronegative patients	197.99	194.2	-2.0	309.40	56.27

PD: peritoneal dialysis; HD: hemodialysis; SUS: Unified Health System; PH: Private Healthcare

According to the Brazilian legislation on PH, the operator or benefits administrator is responsible for informing the contracting party of the main characteristics of the contract to which they are bound, such as type of contract, termination rules, and rules for calculating and applying adjustments^4^. This generates disparities between the amount paid for RRT by the various PH operators. For this reason, when comparing with the SUS, we utilize the most common PH plan in our region.

## Discussion

Our evaluation of PD and HD absorption costing from the point of view of the service provider and the comparison of the results with the funds transferred from the SUS and SS revealed a cost constraints.

PD differs from HD in a number of ways, especially logistically. For example, PD is performed at home and requires little structure within the clinic, whereas HD is performed in a dialysis clinic, which requires more elaborate logistics. In addition to all the logistical data, PD requires fewer staff; one doctor and one nurse can assist 50 patients. In HD, one nurse technician is needed for every six patients, and one nurse and one doctor for every 50 patients. In HD, there are still additional costs such as patient food and transportation, as shown in the list of variables in our study^16^.

The main limitation of the present study was it did not take into account the costs of infectious complications associated with DT, for which the private service provider is responsible. It is worth noting that the Brazilian public (SUS) and private systems (PH) do not cover these expenses, so the cost to the service provider may have been underestimated. Another limitation is that we did not separate the costs of RRT for patients with vascular accesses through arteriovenous fistulas and catheters, which may have different costs.

To the best of our knowledge, the issue of dialysis payment adjustment has not been addressed in Brazilian studies. The adjustment of funds transferred by the SUS and PH in Brazil is not based on studies performed with private service providers, which make up 80% of dialysis centers in Brazil^12^^,^ 19.

As previously reported internationally and in Brazil, we observed an increase in the number of patients that need dialysis over the study period^1^^,^ 2.

Table 1, which takes into account the TEvEP, shows that the costs are allocated as expected. In Tables 2 and 3, other costs were incorporated, and lastly, Table 4 shows the initial calculations that led to a mathematical constant, shown in Table 5, which lists the DT values according to the service provider (see supplementary material for calculations). In Table 5, the fund transfers made by the SUS, when compared to the mean costs of the service provider, were negative for PD (-29.8%), for HD in seronegative patients (-2.0%) and hepatitis B patients (-269.7%). Only hepatitis C seropositive patients on HD were sufficiently funded (+10.2%).

A different result was found for the fund transfers made by SS. For this analysis, only one private healthcare plan was considered, which is the one with the largest number of DT (80%) patients in the considered setting. The funds transferred in this case were positive for PD (+48.26%), for hepatitis C seropositive patients on HD (+64.54%), and for seronegative patients on HD (+56.27%). However, for hepatitis B patients, the funds transferred were insufficient (-50.21%), but still much better (219.5%) than the funds transferred by the public provider (SUS).

International studies comparing private DT providers to the public system provider have not been found in the literature for comparison. In Brazilian studies, the provider is the country's public healthcare system (SUS), and these studies typically compare therapies using different methods, such as direct cost, utility cost, cost-effectiveness, cost-benefit, etc. A cross-sectional study conducted in Italy^20^ analyzed the cost of PD from the point of view of the National Italian Health Service and found that automated PD (APD) had higher costs than CAPD. From the perspective of the UK public system, a study^21^ comparing PD with HD showed that PD cost less to the public system. Another study^22^ evaluating only the cost of HD for the public system in Jordan, a country with a low gross domestic product (GDP), showed that HD had a high cost and concluded that other dialysis modalities should be considered.

Several studies have been conducted with data from the United States Renal Data System (USRDS) database. One such study, a cohort comparing dialysis modalities funded by Medicare, showed that patients on home-based modalities had the lowest cost to the system^10^. Also from the public health point of view, in a cohort followed for ten years in Singapore, it was concluded that starting CAPD was more cost-effective^23^. In Taiwan, a large cohort study^24^ also comparing dialysis modalities using the government database showed that PD was more cost-effective. In a Swedish healthcare system, the mean annual costs were ~50% higher for patients on HD than for those on PD. Compared with the general population, costs were substantially elevated in all groups, from 4-fold in patients with CKD to 11-, 29- and 45-fold higher in transplanted patients and patients on PD and HF, respectively^25^. From the point of view of the public healthcare system, a Korean study^26^ reported findings in agreement to the Swedish study, in 2017.

It is worth noting that, as published^9^, cost analyses from the perspective of the service provider make the data more consistent, transparent, and comparable. This is in accordance with the Brazilian MoH, which recommends using the absorption costing method by the service provider adopted by the National Cost Management Program (*Programa Nacional de Gestão de Custos*). This method was chosen because it is easy to apply and is the most widely used for internal administration of institutions linked to the SUS^15^.

In Brazil, cost studies from the perspective of the public payer (SUS) have been published since 1990^13^. Later studies have been conducted under a theoretical perspective^27^ or again from the SUS perspective^1^^,^ 14 ^,^ 28. A prospective cohort study^11^ evaluating the RRT financial impact on the SUS and SS concluded that after the first two years of RRT, kidney Tx presented lower costs than HD or PD. Another SUS study^19^ estimating the costs of CKD attributable to diabetes concluded that diabetic patients accounted for 22% of CKD and dialysis costs. There are no studies from the perspective of the service providers in Brazil.

Constraints on HD and PD costs revealed by this absorption costing study need to be identified, interpreted, and eventually adjusted/corrected. There is a clear "bidirectional" imbalance of funds transferred from public (SUS) and private (PH) payers to the service provider. The "top-down" funds transferred were mainly negative whereas the "bottom-up" funds transferred were mainly positive. How can this imbalance be justified? We believe that the lower transfer of SUS is due to the lack of studies that assess the real cost of procedures (top down). The funds coming from SUS, in the case of RRT, are calculated based on theoretical estimates (bottom-up), which, as we can see, do not correspond to reality. In addition, we live in a country of continental proportions and to achieve an equal treatment of all regions with regard to payment for services provided the subsidy needs to increase. Add to this, the frequent adjustments of inputs, inflation, and the numerous taxes paid by service providers. All these factors lead to a scenario of cost constraints for service providers, mostly small private clinics.

Only hepatitis C seropositive patients on HD receive sufficient SUS funds (+10.2%). This contrasts with services from PH, in which only hepatitis B seropositive patients were insufficiently funded (-50.21%), but still much better than the funds transferred by the public provider (SUS), -219.5%. What could be the reasons for these two findings, apart from the lack of cost studies that adequately demonstrate the value of the procedures? Hepatitis C patients cannot re-use dialysis materials, which increases the costs of treatment, but they also do not need an exclusive nursing technician or an exclusive room, unlike patients with hepatitis B who require material disposal, need an exclusive nursing technician, and an exclusive room.

It is interesting to note that the funds transferred to PD were negative (-29.8%) from "top-down" by SUS and positive by SS (+48.26%). This significant difference must be due to differences in the way PD therapy is considered. One can speculate that from the SUS perspective PD is a home therapy without many of the costs associated with HD and that it functions like an automated production line in a factory. This is supported by looking at how much they pay (supplies produced and delivered, monthly blood tests and medical services) and to whom most of the payment goes (the manufacturers of PD supplies and those who deliver the goods to the patient at home). It is known that operating costs of PD clinics (much lower than HD clinics, but include utility costs like electricity, water, space rental, telephone line, mobile phone, internet, etc., and costs with the medical team, phone calls and/or telemedicine appointments) are barely recognized in the SUS system.

Other issues involving the low uptake of PD in Brazil, and even in other parts of the world, lie beyond cost. In 2014, Abensur stated that in addition to the low profit margin, the training of nephrologists in this modality is inadequate, and as PD has a lower technique survival, the low indication of this therapy cannot keep enough patients in PD. This leads to a vicious circle, in which the small number of PD patients is not enough for adequately train the professionals, who in turn are less likely to indicate the modality^29^. In line with this observation, Picoli states that RRT consumes 2-5% of general health care expenses in countries where dialysis is available without restrictions^30^. As demonstrated in our study, dialysis costs can be calculated in different ways. We believe that there is not only one determining factor, as countries with different per capita incomes may have similar rates of PD penetration, such as Brazil and the EU^31^. Also, the public policy systems are not determinant of such data. An example is Costa Rica, where the rate of renal transplantation and PD is proportionally higher than in Brazil despite a similar health policy^32^. The type of RRT reimbursement also does not determine the amount, as the reimbursement for each RRT component, by package or by value, does not determine different penetration rates in different countries^30^.

The current trend of the "pay per performance" method can also be discussed. In this system, the funding is allocated according to the quality of the dialysis treatment for each individual patient, in each individual clinic. The funding is based on the "value" created for each patient, and how do you create "value" for the dialysis patient? DT value = patient outcomes divided by treatment costs. However, if we are to follow this method, we must have adequate calculations of the actual costs of the dialysis procedure, which is what we found in our study.

In conclusion, this study is the first to evaluate absorption costing from the perspective of the service provider in Brazil. This study provides governments with valuable and adequate information that may allow DT to adjust the financing of health services, avoiding and loss of money for clinics. This will allow for more comprehensive and better treatment of patients.
